# A scoping review of research literature on eating and body image for transgender and nonbinary youth

**DOI:** 10.1186/s40337-023-00853-5

**Published:** 2023-09-22

**Authors:** Katie Heiden-Rootes, Whitney Linsenmeyer, Samantha Levine, Mark Oliveras, Miriam Joseph

**Affiliations:** 1 Department of Family and Community Medicine, School of Medicine, Saint Louis University, 3700 Lindell Blvd., Ste 1100, St. Louis, MO 63108 USA; 2 Department of Nutrition and Dietetics, College of Health Sciences, Saint Louis University, St. Louis, MO USA; 3 University Libraries, Saint Louis University, St. Louis, MO USA

**Keywords:** Eating, Body image, Disordered eating, Transgender, Nonbinary, Youth, Young adult, Adolescent

## Abstract

**Background:**

Transgender and nonbinary (TGNB) adolescents and young adults are underrepresented in the literature on eating disorders and body image-related problems, despite increased mental health disparities and emerging research showing high associations between gender dysphoria, body image, and eating disorders among TGNB youth.

**Aims:**

The scoping review was designed to critically examine the research on TGNB adolescents and young adults who experience eating and body image related problems as well as clinical studies on treatment approaches and effectiveness.

**Method:**

Preferred Reporting Items for Systematic Reviews and Meta-Analyses extension for Scoping Reviews (PRISMA-ScR) was used for reporting this scoping review. The electronic databases of MEDLINE and PsychInfo were used for searching subject terms. Inclusion criteria for studies required the quantitative measurement or qualitative exploration of body image or eating for transgender minor children, adolescents, or young adult samples (18 to 25 years old) and address differences in eating/body-related problems by age. The relevant data was extracted and narratively summarized.

**Results:**

49 studies were identified, data extracted, and analyzed. Increased prevalence of eating disorders and body image problems were identified for TGNB youth. Body-gender congruence through gender affirming social and medical interventions (e.g., hormone therapy) were noted as significant for alleviating body image problems and facilitating eating disorder treatment. Family and social factors were not well understood in the literature and a need for increased study of TGNB youth from varied racial/ethnic, neurodiverse, and within specific identities (e.g., nonbinary) and families and cultural contexts is still needed.

**Conclusions:**

Future research should consider the use of developmental and family theories for guiding inclusion of salient social factors influencing eating patterns, body image, and treatment outcomes. In addition, more studies are needed with those from minoritized racial and ethnic groups, neurodiversity, and varied gender identities (e.g., nonbinary and gender queer) for identifying important differences.

## Abstract (highlights)

Forty-nine published research studies with transgender and nonbinary (TGNB) adolescents and young adults on eating patterns and body image were identified, summarized, and critically examined. Eating disorders and body image problems are more prevalent with TGNB adolescents and young adults, as compared to their non-transgender peers. More research is needed TGNB minor children, adolescents, and young adults as separate groups and as part of family and social settings where they may experience rejection and delays in seeking and accessing treatment.

## Background

Adolescence and emerging adulthood (here forward called youth) are marked by both increased autonomy, development of a sense of self, reliance on peers [1], and increased risk of mental health challenges including eating disorders and negative body image [2, 3]. A growing number of youth are openly identifying as transgender and nonbinary (TGNB; i.e., gender identity differs from sex-assigned-at-birth, inclusive of nonbinary, gender queer, and other genders) [4] and commonly experience eating disorders and body image problems [5, 6]. There is emerging evidence that eating disorders and body image are related to both gender dysphoria [5] and social factors like family acceptance [6] and bullying by peers [7]. Understanding the influence of external stressors on TGNB youth development, gender dysphoria, and their association with eating disorders and body image problems is vital for the creation of effective treatment.

### Minority stress during youth development: a guiding theory

TGNB youth experience minority stressors during critical periods of development. Minority Stress Theory (MST) was originally created to explain the health disparities of gay, lesbian, and bisexual adults [8], and has since been expanded and nuanced to consider the experiences of TGNB youth in the context of development and family [7]. Health disparities were originally posited to be a product of external and internal stressors [9]. External stressors for youth include stigma, rejection, bullying, and violence by peers, dating partners, and in school settings. The external can become internal stressors of self-rejection (i.e., internalized transphobia), increased gender dysphoria (e.g., psychological distress associated with gender identity not matching the body/sex-assigned-at-birth), expectations for rejection by others [10], and the need to conceal TGNB identities and gender expression, including in their families while growing up (Author et al., under review). Increased external and internal stressors, as MST posited [11], can lead to poor physical and mental health and diminished academic performance for TGNB youth [12, 13].

MST also identifies several protective factors: social and family acceptance, support for a gender affirmation through social changes (e.g., name and pronoun changes, gender marker), access to gender affirming medical treatments (e.g., puberty blockers, hormones, surgery, affirming psychotherapy and treatment) [9, 11], and positive teacher relationships [13]. Family and social acceptance from a variety of sources are protective factors for TGNB youth because they may reduce, and in some cases eliminate, gender dysphoria because families, peers, and schools are allowing and supporting TGNB youth to become gender congruent (i.e., where gender expression and name and pronouns match gender identity) [14]. This is particularly salient for TGNB youth who experience significant body image concerns during puberty due to the development of secondary sexual characteristics (e.g., changing voice, development of chest tissues, etc.) that do not align with their gender identity. This may be a source of significant gender dysphoria requiring caregiver consent and financial support to obtain gender affirming healthcare services such as puberty blockers and hormone therapy [9]. MST can also be used to understand compounding impacts of having more than one marginalized identity, including TGNB youth from minoritized racial groups [15].

In this scoping review, MST was utilized as a framework for identifying significant factors contributing to outcomes for varied ages and differently marginalized subgroups (e.g., TGNB people from marginalized racial and ethnic groups). MST provides a critical lens for the review analysis including types of methods employed, the language used in describing TGNB youth, and the treatment approach and outcomes of TGNB youth. External stressors are experienced across family, school, and healthcare settings for TGNB youth. Thus, MST guides this review to reflexively consider what factors are being considered for TGNB youth that may differ from TGNB adults or other groups experiencing eating disorder and body image problems.

### Current study

MST posits that TGNB youth health outcomes will vary based on external and internal stressors, which are further influenced by mental health comorbidities, age, family and social acceptance, racial/ethnic context, and access to gender affirming healthcare and treatment. MST offers a lens for examining the literature on TGNB youth with disordered eating and body image related problems. This scoping review aimed to answer four research questions: (1) What methodologies are being used to study eating and body image related problems among TGNB youth? (2) What are the risks and protective factors for eating and body image related problems for TGNB youth? (3) Who is being included and excluded in the TGNB youth samples of studies on eating and body image related problems? (4) What are the empirically supported treatments for eating and body image problems for TGNB youth? The focus of this paper is youth only. TGNB adult literature is detailed in another scoping review (Authors, et al., accepted).

Current literature reviews on TGNB youth and eating disorders span broad sexual and gender minority populations and ages [16, 17] or provide a narrow focus on diagnosis rates and symptom presentation [16, 18, 19]. We conducted a scoping review to critically examine the breadth of research about TGNB youth who experience eating and body image problems. We aimed to incorporate studies addressing treatment and intervention, mental health comorbidities, body image, gender dysphoria, food security, and general eating patterns that are not necessarily disordered in nature. Given the theorized use of disordered eating behaviors to attain a body size or shape that is an attempt to meet gendered appearance ideals [18], we include studies with both eating and body image variables.

## Method

This scoping literature review adhered to the Preferred Reporting Items for Systematic Reviews and Meta-Analyses extension for Scoping Reviews (PRISMA-ScR) guidelines in the search, review, and reporting processes [20] and included both adults and youth during first searches. This article follows the same search procedures as outlined in the companion TGNB adult manuscript (Author et al., accepted). The search procedures are retained below for review.

The search strategy was developed through initial meetings and consultation between the first author and the university librarian (last author, MJ) in the fall of 2020. Preliminary searches were conducted using the OVID interface of possible databases including *MEDLINE, PsychINFO, CINAHL: Cumulative Index to Nursing and Allied Health Literature, Cochrane Database of Systemic Reviews, Social work Abstracts, Social Services Abstracts,* and *Sociological Abstracts* to identify potential articles about transgender adults and eating, body, and weight related problems. The second author (WL) was consulted based on her expertise in nutrition and dietetics to review initial searches for relevant articles.

**Fig. 1:**
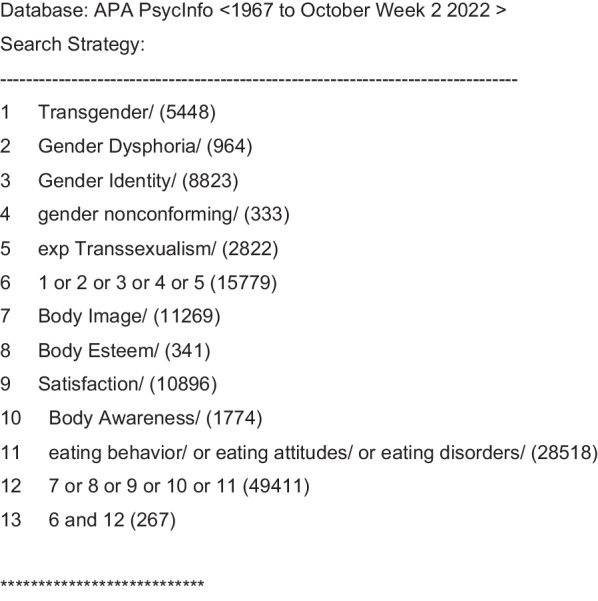
Example search string example for literature review

The preliminary searches demonstrated two databases—*MEDLINE* and *PsychINFO*—were superior for identifying relevant articles. To search the databases, we identified official search terms (i.e., internal vocabulary) in the databases through the OVID interface (example of the search string can be found in Fig. 1). In MEDLINE the search terms—*transgender persons, gender identity, transsexualism, gender dysphoria, body image, body dissatisfaction, self-concept, feeding behavior, anorexia nervosa, binge eating,* and *bulimia nervosa—*were used. The trans/gender terms and body/eating terms were then searched together for identifying articles where both subjects were categorized*.* A similar process was used for PsychINFO with the following subject terms—*transgender, gender dysphoria, gender identity, gender nonconforming, transsexualism, body image, body esteem, body satisfaction, body dissatisfaction, body awareness,* and *eating behavior or attitudes or disorders. Eating behavior or attitudes or disorders* subject terms included anorexia, bulimia nervosa, and binge eating disorders. No limits were set by date of publication in order to capture the changing theories and findings in the field up to current literature in December 2022.

### Inclusion and exclusion criteria

Articles included in this review met the following criteria: (1) published in peer review journals (including online advance publications); (2) published in English language by December 2022; (3) described qualitative or quantitative empirical research (including case reports and case studies); (4) sample of transgender youth (inclusive of transmasculine, transfeminine nonbinary, and gender expansive or questioning); and (5) addressed review questions about eating behavior and body image including those addressing treatment and intervention. “Youth” was defined as minor child to adolescent (< 18 years old) and emerging adulthood (18 to 25 years old). Given the critical period of development cognitively, identity formation, and risks for mental and physical health among young adults under the age of 25, we elected to include these emerging adults in the youth subpopulation [21]. TGNB adult-only articles were separated out and are reported on in a separate manuscript (Author et al., under review).

The following types of studies were excluded from the review: book chapters; review articles; editorial commentaries; clinical opinion articles without case or research data; non-English language studies; dissertations; studies where outcomes from transgender participant data were not reported separately from the larger sample; studies that did not include at least one of the following—eating behavior or disorder measurement, body image scale, interview data on eating or body image.

### Review and data analysis process

**Fig. 2:**
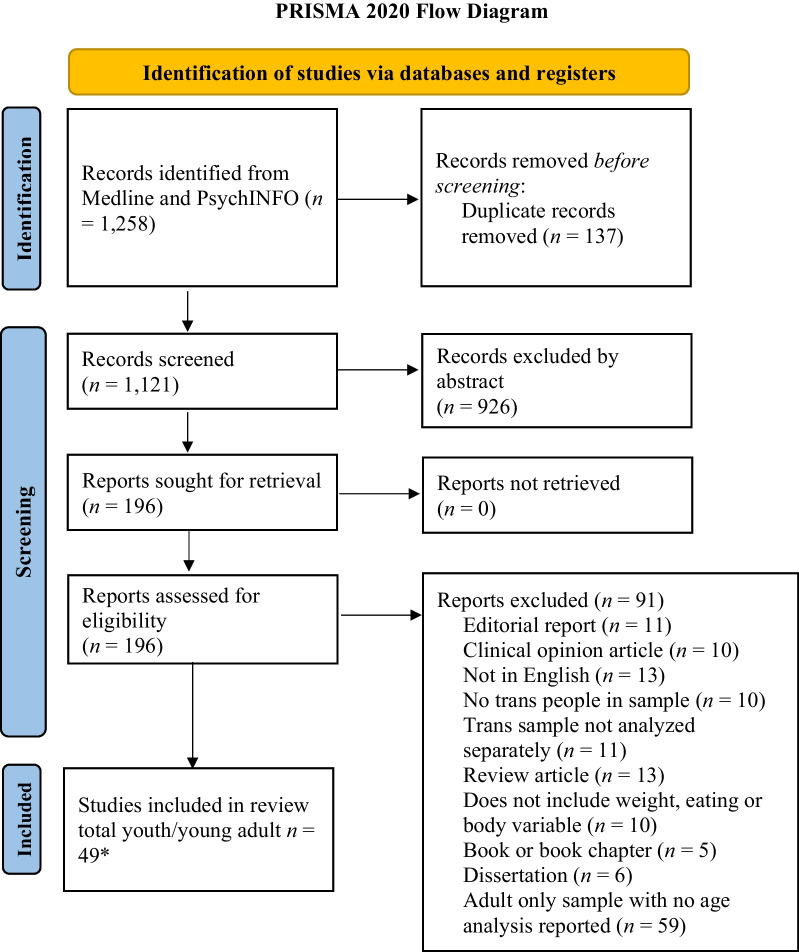
PRISMA 2020 flow diagram of the systematic literature review process. *Note. Some samples included both youth and adults with analysis by age for obtaining separate results based on age/developmental period

The identified articles were uploaded to Covidence©, an online software, for managing duplication removal and then the process of abstract review, full text review, and, finally, data extraction. Duplications (*n* = 136) were removed initially by the software. This was reviewed by the first author to ensure accuracy of the removal. Reviews were completed by three research team members (KHR, WL, SL) and four undergraduate and graduate research assistants. Pairs of authors and research assistants reviewed abstracts based on the inclusion/exclusion criteria. Discrepancies in the reviews were resolved by the first and second authors. Then, full text reviews were completed by the second and third authors and the research assistants. Again, discrepancies were resolved by the first and second authors. Data extraction was completed by the second, third, and fourth authors. Finally, one case control study article was removed at data extraction as two reviewers (KHR, SL) agreed that the study did not meet our inclusion criteria as transgender people were the subject of the work, but not the participants in the study [22]. The PRISMA figure (see Fig. 2) outlines the course of the review and article selection and extraction.

Data was then downloaded from Covidence© to a spreadsheet developed by the first author where the sample characteristics, guiding theories, definitions of ‘transgender’ and/or language about gender, measurements, funding sources, limitations, bias, and other commentary were noted. The third and fourth authors led the data movement to the spreadsheet. Then analysis of the data followed three modalities. First, studies were separated by age to create a youth (children to emerging adulthood, age 25) and adult (adult-only samples) tables for separate analysis. Then study methodology (quantitative, qualitative, and case report) was analyzed by the first and third authors identifying and quantifying types of methodology, sample size and demographics, measurement use, and geographic location. We also to identify limitations inherent in current methodologies for drawing meaningful conclusions. Variable outcomes (eating disorders, body image) were analyzed by the second and fourth authors. The results were then narratively summarized. Variable outcomes were reported based on divergent and consistent findings across studies. In addition, unique findings were noted for subsamples, if appropriate.

Finally, overarching bias and limitations in the studies were summarized. To assess rigor and potential bias in studies, the authors used four items from the STrengthening the Reporting of OBservational Studies in Epidemiology (STROBE) checklist [23] and the Standards for Reporting Qualitative Research (SRQR) checklist [24] as previously used in systematic reviews with women with minoritized sexual identities [25]. This allowed for a critical review of study biases. The items were: 1) The authors describe the eligibility criteria and the sources, methods, and rationale of participant selection; 2) The authors describe the characteristics of study participants (coded “yes” if the authors provided information about age, race/ethnicity, and socioeconomic status); 3) The authors describe and provide a rationale for their quantitative or qualitative analytic methods; and 4) The authors discuss the limitations of the study, including sources of potential bias or imprecision. This allowed for the potential for bias to be assessed after examining data extraction of the results and was completed by the first and second author. This manuscript will only report on the youth and young adult articles as defined.

## Results

The review yielded 49 articles (see Table 1). The studies represent adolescent and emerging adulthood stages within their youth samples. Of those 49 studies, 25 studies focused exclusively on emerging adults, 15 included minor/adolescent age participants, and the final 9 included samples with a combination of adolescent and emerging adult. The emerging adult only samples were largely college student surveys or case reports from eating disorder treatment clinics. The minor/adolescent samples were often surveys completed through high schools or case reports in eating disorder treatment clinics. Most studies focused on eating disorders or patterns of eating for weight management (*n* = 36 studies), while the remaining studies covered body image, body satisfaction, and body-gender congruence. Finally, one study with minor youth aimed to develop a scale for eating disorders with TGNB youth [26]. Findings from the minor youth only studies outline comorbidities of eating disorders and body image with mental health disorders, neural diversity (i.e., autism and attention deficit disorder), and gender dysphoria.

**Table 1:** Youth articles retained for review

References	Sample size	Eating disorder or body image focus	Sample demographics	Study design	Quantitative measures	Qualitative interview domains	Findings	Country of origin
Avila et al. [26]	n = 106	Eating disorders	64 youth transmasculine, 30 youth transfeminine, and 12 nonbinary participants. Adolescents	Cross-sectional	Eating Disorders Examination Questionnaire, two questions developed about frequency of intentional weight manipulation behaviors for gender-affirming purposes	N/A	A majority (63%) of participants reported intentional weight manipulation with the goal of altering their body type to match their gender identity	United States
Beaty et al. [55]	n = 1	Eating disorder	27-year-old trans woman patient	Case study	weight, kcal intake, phosphorous level, and phosphorus supplementation on days 1, 4, 6, 9, 10, 11, 12, 13, 71, and 92 of hospitalization	N/A	High-dose estrogen can negatively impact phosphorus levels by increasing urinary extortion of phosphorus, and thus the hypophosphatemia described in this patient was a result of increased renal losses as a result of supraphysiologic oral estrogen state	United States
Becerra-Culqui et al. [47]	n = 27,633	Eating disorder	588 transfeminine and 745 transmasculine children/youth, with cisgender referent matched controls for each TGNC individual. Racial diversity present	Cross-sectional	Age and demographics; EHR analysis that assessed the presence of various mental health diagnoses and transgender status	N/A	Eating disorder diagnoses were found to be more prevalent among transmasculine and transfeminine children compared to the reference cisgender children	United States
Becker et al. [81]	n = 202	Body image	62 adolescent trans women, 20 adolescent trans women, 50 adult trans men, and 70 adult trans women	Cross-sectional	Body Image Assessment Questionnaire: “attractiveness/self-confidence” scale, “accentuation of body appearance” scale, “insecurity/concern” scale, and “sexual-physical discomfort” scale	N/A	Adolescents had a less favorable body image compared to adults on all four scales. Transgender participants who already received medical interventions reported a less impaired body image	Germany
Becker et al. [82]	n = 864	Body image	135 trans men adults, 115 trans women adults, 235 cisgender female adult controls, and 379 cisgender male controls	Cross-sectional	Age, Hamburg Body Drawing Scale	N/A	The transgender participants reported lower satisfaction with their overall appearance. Trans men presented more extreme dissatisfaction with body features typically associated with the female sex, while trans women presented extreme dissatisfaction with features including body hair and genitalia	Netherlands, Germany, and Norway
Burke et al. [39]	n = 145,379	Eating disorder	2,983 gender minority participants and 142,396 cisgender participants. Racial diversity present	Cross-sectional	Five-item SCOFF eating disorder questionnaire	N/A	Multiracial Indigenous and Hispanic/Latinx persons (and other doubly marginalized groups) had the greatest prevalence estimates of increased ED pathology. ED prevalence was greater than expected based on the observed prevalence estimates in their respective monoracial groups	United States
Cibich and Wade [56]	N = 1	Eating Disorder	One adolescent trans man patient	Case report	BMI, number of objective binges, subjective binges, and purges per week, Eating Disorder Examination Questionnaire, Depression Anxiety and Stress Scales-21, Clinical Impairment Assessment	None	Patient viewed restrictive eating as a way to de-emphasize the feminine fat distribution and obtain a masculine physique. Client reduced ED behaviors and reduced the frequency in which he compared himself to masculine males, and reduced his global eating disorder score though cognitive behavioral therapy	Australia
Coleman and Cesnik [50]	N = 2	Body Image	Case 1: 23-year old man Case 2: 23-year-old male	Case study	Tennessee Self-Concept Scale, Minnesota Multiphasic Personality Inventory, Beck Depression Inventory, Hamilton Depression Scale, Hamilton Anxiety Scale, Brief Psychiatric Rating Scale	N/A	Lithium carbonate therapy may alleviate the intense obsessional thinking in some cases of gender dysphoria. There may be a biomedical correlate and associated psychopathology to the obsessional drive toward body alteration through hormonal and surgical means	United States
Cooney et al. [57]	N = 1	Eating Disorder	13-year-old trans man	Case study	BMI, hormone levels	N/A	The patient became motivated to lose weight after initial weight loss resulted in decrease in pre-op chest size. Gender identity may be an important variable to assess when working with adolescents as gender dysphoria may contribute to psychosocial struggles that impact weight loss	Canada
Couturier et al. [58]	N = 2	Eating disorder	16-year-old Caucasian trans woman and 13-year-old Caucasian trans man	Case study	None noted	N/A	As eating disorder symptoms lessened, the desire to change secondary sex characteristics became evident. The increased expression of gender dysphoria was associated with a reduction in eating disorder behaviors, possibly driven by the accentuation of secondary sex characteristics as weight restoration progressed	Canada
Cusack and Galupo [27]	N = 194	Body image and eating disorder	Total 194 nonbinary adults. 122 assigned female, 61 assigned male, 4 assigned intersex, and 7 who did not respond. Racial diversity present	Cross sectional	Body Checking Questionnaire (BCQ); Body Appreciation Scale (BAS) is a positive measure of body image; Eating Disorder Examination-Questionnaire (EDE-Q)	Open-ended question about other body checking behaviors that were not reflected in the BCQ	Body checking predicted eating disorder pathology, and body image significantly improved the model. Gender congruence did not additional variance in predicting eating pathology	United States
Diemer et al. [45]	N = 289,024	Eating Disorder	479 (.17%) transgender students, 5977 (2.07%) cisgender, sexual minority male students, 1662 (.58%) cisgender unsure men, 91,599 (31.69%) cisgender heterosexual men, 9445 (3.27%) cisgender sexual minority women, 3395 (1.17%) cisgender unsure women, and 176,467 (61.06%) cisgender heterosexual women Age and racial diversity present	Cross-sectional	Questionnaire asking about sexual orientation and gender identity, past-year ED diagnosis and behaviors including vomiting, laxative pill use, and diet pill use were assessed by questions with yes/no answers. As well as race, cigarette use, binge drinking, stress levels, athletic participation, and fraternity/ sorority membership	N/A	Transgender students had treated odds of past-year eating disorder diagnosis, past-month diet pill use, and past-month vomiting or laxative use compared to cisgender, heterosexual women. Compared to heterosexual or sexual minority transgender students, transgender students who were unsure of their sexual orientation had significantly higher rates of past-year ED diagnosis, past-month vomiting or laxative use, and past-month use of diet pills	United States
Donaldson et al. [30]	N = 5	Eating Disorder	Participant 1: 15-year-old transgender female Participant 2: 22-year-old who presented to ED clinic at age 17 and identified as a cisgender female and then asserted gender queer Participant 3: 19-year-old transgender man Participant 4: 13-year-old who first identified as a transgender male at age 10 and then asserted gender nonconformity at age 13 Participant 5: 14-year-old transgender male who asserted gender identity at age 5	Case Series	BMI	Screening questions developed by the eating disorder and gender clinics	Findings suggest that gender nonconforming youth may turn to maladaptive behaviors to change their bodies as a result of the absence of timely gender dysphoria management. This study highlights several barriers to timely care, including parental refusal to support gender identity, parental rejection of gender-affirming treatment, and lack of timely referral to gender or ED clinic	United States
Duffy et al. [38]	N = 71	Eating Disorder	19 transgender women, 22 transgender men, 27 nonbinary people, 3 people with another gender identity. Racial diversity present	Measurement Testing, Cross-sectional	Eating Disorder Examination Questionnaire Short Form (EDE-QS)	N/A	The EDE-QS demonstrated internal consistency and correlated significantly with indices of disordered eating and body image. Mean values for gender identity groups did not significantly differ from one another	United States
Duffy et al. [32]	N = 84	Eating Disorder	6 women, 30 men, and 48 nonbinary participants Adults, racial diversity present	Qualitative survey data	Demographics	Online qualitative questionnaire addressing psychiatric history, ED and treatment history, and experiences as a transgender person	One identified theme of this study regards the role of the body in eating disorder treatment, including the role of the physical body as the cause of the eating disorder, which was highlighted by about one third of participants. Others explained that the issue of body image is more complex for transgender clients and therefore eating disorder treatment needs to focus on more than a positive body image, and rather include transition in the eating disorder recovery process	United States, Europe, and Canada
Duffy et al. [46]	N = 365,749	Eating Disorder	237,844 cisgender females, 127,227 cisgender males, and 678 transgender participants Adults, racial diversity present	Cross-sectional	Survey questions covered topics including gender identity, past-year eating disorder status, history of non-suicidal self-injury, and history of suicidal ideation and attempts	N/A	About 1.5% of the overall sample reported past-year eating disorder diagnosis or treatment, with rates higher for transgender participants than for cisgender participants. Rates of past-year NSSI, suicidal ideation, and suicide attempts were highest among transgender individuals with eating disorders (EDs)	United States
Ewan et al. [48]	N = 1	Eating Disorder	19-year-old Hispanic trans woman patient	Case report	BMI, medical labs including hormone levels	Medical history, eating disorder behaviors, motivations for weight loss, and course of treatment	The patient’s desire to lose weight and onset of disordered eating behaviors coincided with her decision to transition socially. She began testosterone suppressants for medical gender transition to aid in eating disorder recovery as her desire to appear more feminine was inextricably linked to her disordered eating behaviors	United States
Feder et al. [5]	N = 97	Eating Disorders	60 patients who were assigned female at birth (61.9%) and 37 patients who were assigned male at birth (38.1%). 58 participants identified as male and 2 identified as gender fluid Youths	Retrospective chart review	Patient demographics including sex, gender identity, sexuality, age, height, weight, body mass index at assessment and over the course of treatment; assessment for DSM-5 criteria for Gender dysphoria and eating disorders; presence of co-morbid psychiatric diagnoses, pre-existing medical conditions, medications, medical and psychiatric admissions, diagnostic evaluations, and ED and GD-relevant treatments	N/A	95% of the sample studied endorsed body dysmorphia and gender dysphoria. Transgender men were found to have a 19 × increased risk of having a restrictive ED compared to cisgender females in the population, while transgender women were found to have a 10 × increased risk compared to cisgender females	Canada
Ferrucci et al. [83]	N = 10,415	Eating Disorder	10,415 transgender people	Cross-sectional	ICD 10 codes for eating disorders. Covariates included age, region of medical service within the United States, relationship to plan-holder, sex reported on claims, and type of insurance coverage	N/A	Unspecified feeding and eating disorders were the most diagnosed eating disorders, followed by anorexia nervosa, other specified feeding and eating disorders, bulimia nervosa, binge eating disorder, and avoidant restrictive feeding and intake disorder. Those diagnosed with any eating disorder were more likely to be young, reported as female on claims	United States
Gordon et al. [29]	N = 21	Body image and eating disorder	11 participants who identified as female, 7 as transgender, 1 as transsexual, 1 as genderqueer, and 1 as demi-girl Adults, racial diversity present	Qualitative	N/A	Interviews covered body satisfaction, needs for gender affirming body change, stress and coping, social influences on feelings about appearance, and past or current experiences with weight and shape control behaviors, demographic characteristics, body image, and weight and shape control information	Four main themes: (1) Gender socialization processes and the development of femininity ideals; (2) Experiences of stigma and discrimination; (3) Biological processes; and (4) Resilience processes. Narratives suggest that some parents or peers might manage their anxieties or prejudices about gender identity by channeling them into more socially acceptable narratives about weight gain and fitness	United States
Grammer et al. [37]	N = 8,531	Eating disorder	230 transgender students, 8,301 cisgender students. Racial diversity present	Cross-sectional	the self-report Stanford-Washington University ED Screen (SWED) (Graham et al., 2019)	N/A	Cisgender female students and GD students reported significantly greater odds of a probable ED diagnosis and greater elevations in weight and shape concerns compared to cisgender male students. Some SD students and GD students who met criteria for probable EDs were also more likely to report chronic ED symptoms and probable comorbid psychiatric diagnoses compared to heterosexual students and cisgender males, respectively	United States
Guss et al. [65]	N = 2,473	Eating disorder	67 transgender participants, 1,117 cisgender males, and 1,289 cisgender females Age and racial diversity present	Cross-sectional	age, self-reported weight, height, weight management behaviors	N/A	Transgender participants had the highest prevalence of diet pill use and laxative use	United States
Hartman-Munick et al. [36]	N = 32	Eating disorder	8 transgender women, 13 transgender men, 9 nonbinary adults, and 2 who identified as another gender. Racial diversity present	Qualitative, Inductive	N/A	online forum and focus group questions about experiences with ED	Three major themes emerged from the analysis: (1) Barriers to ED screening/treatment; (2) Complexity of the relationship between EDs and gender dysphoria; (3) Need for provider education in gender affirming care practices for ED screening and treatment	United States
Hepp et al. [54]	N = 2	Eating disorder	2 identical twins with eating disorders. One had GID and one did not	Case study	Demographics, BMI	Relevant medical history	Hypotheses offered about the origins of transgender identity and dysphoria	Switzerland
Himmelstein et al. [41]	N = 9,679	Eating disorder	9,679 LGBTQ teens Racial diversity present	Cross-sectional	Demographic data including BMI, weight-based victimization frequency, frequency of dieting, binge eating, and strategies for weight control on a 4-point scale, Godin Leisure Time Exercise Questionnaire, frequency of difficulty falling asleep, Motivations to Eat Scale	N/A	Weight based victimization associated with adverse eating, dieting, and weight-related health behaviors for sexual and gender minority adolescents	United States
Idrus and Hymans [84]	N = 30	Body image	30 transgender waria adults	Qualitative	N/A	Semi-structured interviews covering body image, shape, and efforts to change their body	Many took contraceptive hormones meant for women as a means of reshaping bodies. They also used various whitening agents to change skin color	India
Kuper et al. [72]	N = 148	Body image	148 youth receiving gender-affirming hormone therapy Gender identity. Male, boy, or guy: 81 (55%), Male spectrum: 9 (8%), Female, girl, or woman: 52 (35%), Female spectrum: 2 (1%), Something else: 3 (2%) Racial diversity present	Cross-sectional	Body Image Scale, Screen for Child Anxiety Related Emotional Disorders, Quick Inventory of Depressive Symptoms; Gender identity, sexual orientation	N/A	Significant decrease in body dissatisfaction, self-reported depressive symptoms, and total anxiety symptoms was observed during the follow-up period. Decreases in generalized, separation, and school-related anxiety symptoms were significant. No change in clinician report of depressive symptoms was found	United States
Lemma [51]	N = 1	Body image	1 trans woman	Case study	N/A	N/A	Transgender people are looking for identity congruence	Not known
Lin et al. [70]	N = 46	body image	13 trans men, 10 trans women, 11 cisgender males, and 12 cisgender females. Adults	Cross-sectional	Beck Depression Index, Defense Style Questionnaire, Klein Sexual Orientation Grid, FMRI	None	Transgender individuals more concerned with body image as a central feature of their identity	Taiwan
Linsenmeyer et al. [67]	N = 164	Eating disorder	128 transgender men, 28 transgender women, and 8 nonbinary people	Cross-sectional	Sick, Control, One Stone, Fat, Food Questionnaire (SCOFF), Adolescent Binge Eating Disorder Questionnaire (ADO-BED), Nine-Item Avoidant/Restrictive Food Intake Disorder Screen (NIAS), and Hunger Vital Sign	Questionnaire about previous ED diagnosis, demographic information	A majority of participants were a healthy weight, while 17.1% were overweight, and 28.0% were obese. An estimated 8.7% reported a previous eating disorder diagnosis. 28.0% screened positive on the SCOFF, 9.1% on the ADO-BED, 75.0% on the NIAS, and 21.2% on the Hunger Vital Sign. Transgender males scored higher on the NIAS than transgender females. Those with a previous eating disorder diagnosis scored significantly higher on the Hunger Vital Sign	
Lipson et al. [66]	N = 65,231	Eating disorder	1,237 gender minority students and 63,994 cisgender students Racial diversity present	Cross-sectional	2 questions to identify gender identity, Patient Health Questionnaire, Generalized anxiety disorder scale, SCOFF screen	N/A	Demonstrated 2 to 4 times higher prevalence of depression, anxiety, eating disorders, self-injury, and suicidality in gender minority sample as compared to cisgender	United States
Manzouri et al. [71]	N = 96	Body image	28 trans men transexuals, 34 cisgender males, and 34 cisgender females Adults	Cross-sectional	FMRI, Body perception task	N/A	Possible biological markers in own-body image network for gender dysphoria in transgender individuals	Sweden
McGuire et al. [43]	N = 90	Body image	31% trans men participants, 37% trans women participants, and 32% other gender participants Adults, racial diversity present	Qualitative	Demographic information, Body Image Scale adapted for transgender individuals	In depth interviews about body satisfaction, eating patterns, and social acceptance	Themes identified how the intersection of gender and body size influenced body image satisfaction. This was also influenced by self and other acceptance	United States, Ireland, and Canada
Murray et al. [52]	N = 2	Eating disorder	1 23-year-old trans woman of Indian origin and 1 24-year-old trans woman of Chinese origin	2 case reports	N/A	Therapy	Masculinity and femininity play a role in eating disorders. For example, ideas of femininity lead to a drive for thinness while masculinity in eating disorders is a drive for more muscle tone	United States
Peterson et al. [85]	N = 96	Body Image	54 trans men participants, 31 trans women participants, and 15 nonbinary/ gender fluid participants Age diversity present	Cross-sectional	Author developed questionnaire regarding interest in weight changes, past drug or alcohol use, history of bullying, history of suicide attempts, and body image concerns	N/A	A desire for weight change, either weight loss or weight gain, was associated with increased likelihood for history of suicide attempts among this sample	United States
Peterson et al. [68]	N = 249	Eating disorder	249 transgender youth seeking hormone therapy. Age diversity present	Cross-sectional	Eating Disorder Examination Questionnaire	N/A	Eating Disorder Examination Questionnaire may be appropriate for use with transgender youth	United States
Pham et al. [86]	N = 3	Eating disorder	1 transgender man and 2 transgender women	Case series	ASD severity, BMI	clinical interviews; medical records review	Transgender youth with ASD may experience diverse features of disordered eating that may or may not be uniquely related to gender dysphoria. Youth with both gender dysphoria and ASD may be at higher risk of negative clinical outcomes because of the additive effects of gender dysphoria and ASD	United States
Pistella et al. [42]	N = 31,609	Eating disorder	358 transgender individuals, 31,251 cisgender individuals; Racial diversity present	Cross sectional	California Healthy Kids Survey (CHKS) with supplementary Physical Health and Nutrition Module (PHMN)	N/A	Transgender students who felt less safe at school, participated in less exercise at school but exercised more overall, and exhibited both healthy eating habits and unhealthy eating habits	United States
Ristori et al. [49]	N = 2	Body image, eating disorder	One 14-year-old assigned male at birth One 16-year-old assigned female at birth	Case study	Gender Identity/Gender Dysphoria Questionnaire for Adolescents and Adults, The Beck Depression Inventory, the Beck Anxiety Inventory, and the Youth Self-Report subscales of Internalizing, Anxiety/ Depression, Social Withdrawal, the Body Uneasiness Test, Multi-Attitude Suicide Tendency Scale, BMI, age	N/A	In both patients, treatment to suppress puberty with gonadotropin-releasing analogues improved psychological functioning and resolved pathological eating behaviors	Italy
Roberts et al. [45]	N = 2,110	Eating disorder	633 transgender men, 63 transgender women, 443 nonbinary adolescents, and 919 cisgender adolescents. Racial diversity present	Cross-sectional	The Eating Pathology Symptoms Inventory (EPSI); Transgender Congruence Scale	N/A	Among gender minority participants, adolescents who experienced greater gender identity congruence reported lower levels of binge eating, cognitive restraint, purging, caloric restriction, and muscle building. Nonbinary/questioning assigned female at birth adolescents endorsed higher caloric restriction than cisgender girls. Transmasculine adolescents engaged in greater caloric restriction than cisgender girls and boys. Transmasculine adolescents engaged in greater muscle building than all other groups	United States
Röder et al. [73]	N = 126	Body image and eating disorder	23 transgender females and 103 transgender males All adolescents	Cross-sectional	KIDSCREEN-27, KIDSCREEN-10, Body Image (weight loss behavior) via one question. Hamburg Body Drawing Scale, Youth Self Report	N/A	Transgender participants had significantly lower Health Related Quality of Life scores compared to norm scores. Transgender males scored significantly lower on poor peer relations than transgender females. 50% of all adolescents in the study reported intention to lose weight or weight-loss behavior,	Germany
Romito et al. [7]	N = 9	Body image and eating disorder	7 adolescents assigned female at birth, 2 assigned male at birth	Case series	demographic survey; questionnaires assessing body image and disordered eating behaviors	Interviewers used open-ended questions to engage participants in a semi-structured discussion about their weight, shape, body, and eating behaviors both before their transition and since their transition	Three themes were described: (1) Disordered eating behaviors aimed to align the body with one’s gender identity; (2) Disordered eating behaviors related to broader mental health concerns (eating behaviors as means of coping with depression etc.); (3) The influence of developmental and social context (parent delayed treatment, romantic partnerships)	United States
Simbar et al. [74]	N = 90	Body Image	31% female participants and 69% male participants Patients with no hormone therapy or surgery: 30, Patients with hormone therapy: 30, Patients with gender reassignment surgery: 30, Adults	Cross sectional	Quality of Life questionnaire and Fisher's Body Image questionnaire	N/A	Surgery significantly improved the quality of life and body image of individuals with gender dysphoria. Body image, quality of life, and physical health were all positively correlated. Authors highlighted the significant social health on the quality of life scale	Iran
Simone et al. [28]	N = 13,584 students	Eating disorder	4,526 cisgender men, 8,820 cisgender women, and 238 transgender or genderqueer participants College students/adults, racial diversity present	Cross sectional	Age, BMI, gender identity, sexual orientation, self-reported mental health diagnoses, stressors, substance use, eating disorders impacting academic education	N/A	Compared to cisgender male participants, cisgender women and transgender or genderqueer participants reported greater odds of past year eating pathology that impaired their academic performance and a self-reported lifetime diagnosis of anorexia nervosa	United States
Simone et al. [34]	N = 5,057	Eating disorder	1205 transgender men, 506 transgender women, 2717 gender queer or gender non-conforming, and 629 gender expansive students. Racial diversity present	Cross-sectional	SCOFF questionnaire	Demographic information	Prevalence of clinically relevant ED risk was highest among gender queer/non-conforming (GQ/NC) students (38.8%), followed by trans women (37.1%), gender expansive students (34.0%), and trans men (34.1%). Prevalence of ED risk was higher among trans men who identified as gay (36.7%), bisexual (40.4%), queer (34.6%), or another sexual orientation (44.6%) relative to heterosexual trans men (23.6%)	United States
Strandjord et al. [60]	N = 1	Eating disorder	A 16-year-old white transgender man	Case study	N/A	N/A	Case report describes adolescents with gender dysphoria where disordered eating was the presenting symptom. The patient also shared an association between a desire to appear less feminine and his eating disorder	United States
Surgenor and Fear [62]	N = 1	Eating disorder	One 25-year-old transgender woman patient. Born in New Zealand and of mixed Fijian-Indian and European ethnicity	Case study	N/A	N/A	Patient presented with an eating disorder associated with transgender identity. She used restricting and purging to obtain a more feminine shape, which she viewed as “success” as a transgender person	New Zealand
Wagner and Stevens [59]	N = 1	Eating disorder	23-year-old white transgender man patient	Case report	None	None	Review of patient diagnosed with anorexia nervosa for 6 years and reluctance to engage in treatment. Saw himself as less masculine because of eating disorder	United States
Watson et al. [6]	N = 923	Eating disorder	Adolescent sample, age and racial diversity present	Cross-sectional	Questions regarding school connectedness, family connectedness, perception of friends caring; Medical outcomes study social support survey, Questions regarding binge eating, lose weight by fasting/diet pills/laxatives/and vomiting	N/A	Higher rates of harassment and discrimination was linked to higher odds of disordered eating behavior such as binge eating, fasting, or vomiting to lose weight, while family and school connectedness and social support had a protective effective against odds of disordered eating	Canada

After the review process, these are the final articles retained for review and used in results summaries

### Who is being included and excluded in the TGNB samples of studies on eating and body image related problems?

Across study types, samples were predominately racially white samples though some larger U.S. studies were racially/ethnically diverse. Some studies defined “transgender” as a broad umbrella term that included nonbinary and genderqueer, while others specifically delineated non-binary or gender queer individuals in the samples they were studying. Only one study explicitly studied only nonbinary youth [27]. Most eating disorder studies identified non-binary or gender queer participants [7, 26–39] yet analyzed them as part of the larger “transgender umbrella” because the subsamples were too small to analyze separately. Only three studies analyzed them as a subsample separate from binary transgender youth [28, 30, 34]. Clinical samples relied on the existence of gender dysphoria or the older diagnosis of gender identity disorder, while all others used the terms transgender, male-to-female, female-to-male, gender minority, gender diverse, and gender nonconformity. The studies with large surveys conducted with colleges and high schools allowed for self-identification as TGNB.

The variety in language made it difficult to determine who, in fact, was in the studies based on gender identity alone. Language is a significant issue in research with TGNB samples as language for describing gender identity continues to evolve culturally and among young people. Researchers may rely on medical terms or terms used by parents that do not match TGNB youth language or experiences leading to pathologizing and misrepresenting the experiences of TGNB youth in empirical literature, see Farley and Kennedy [40] for an example.

A review of bias and limitations across studies showed 12 studies lacked limitations sections and 13 studies were missing acknowledgments of potential biases in the study. About half of the studies were funded (*n* = 26) by a variety of funding sources including federal and internal university funding. Only 12 studies included discussion of theory or a guiding theory. Of the studies that did include theory, six referenced minority stress theory [28, 34, 36, 37, 41, 42], while objectification theory [7], intersectionality [39], queer theory [43], and ecosocial theory and the gender affirmation framework [29] were each mentioned once.

### What methodologies are being used to study eating and body image related problems with TGNB people?

#### Quantitative findings

A total of 28 quantitative studies were identified. All articles retained for this study employed a cross-sectional study design. Body image alone was studied in eight articles with mostly young adult samples. Eating disorders alone were studied across 19 samples with minor youth and young adult samples. The remaining two studies measured both body image and eating disorder variables. Several different measures were used to assess the variables of interest. The most common were Eating Disorder Examination (self-report questionnaire, *n* = 4 studies) and the SCOFF (*n* = 3 studies). Other eating disorder scales used include the Adolescent Binge Eating Disorder Questionnaire, Nine-item Avoidant/Restrictive Food Intake Disorder Scale, Stanford-Washington University Eating Disorder Screen, Eating Pathology Symptoms Inventory, and Motivations to Eat Scale. Body image scales varied widely across studies and is discussed further in the body image section of the results. Finally, the Transgender Congruence Scale was used in two studies [35, 44] for understanding congruence between body appearance and gender identity.

The sample sizes of the cross-sectional studies were well-distributed. One study included fewer than 50 participants, six had sample sizes between 50 and 100, six had sample sizes between 101–250, two had sample sizes between 251–500, one had a sample size between 501–1000, four had sample sizes between 1001–5000, and eight had sample sizes greater than 5000. Of the cross-sectional studies, only two pairs drew from the same sample populations. Two studies utilized results from the American College Health Association—National College Health Assessment, a national survey of college students collected between Fall 2008 and Fall 2011 [45, 46]. However, these two studies selected differing sub-samples and thus resulted in different sample sizes. Similarly, two studies were conducted as part of the Study of Transition, Outcomes, and Gender (STRONG) cohort, but their studies include differing sub-samples of the population [44, 47].

Most of the studies originated from within the United States (*n* = 22). The samples were drawn from two primary sources: clinical samples (*n* = 12) or college or high school student surveys (*n* = 8). Finally, most of the quantitative studies included primarily racially White samples, while six included large racially diverse populations with between 50 and 70% white participants [35, 37–39, 42, 47]. Finally, one study was a retrospective chart review with a mixed method design though relied heavily on assessment scales to analyze 60 patients sex- assigned-at-birth female and 27 patients sex-assigned-at-birth male [5]. This Canadian study included participants ages 12–18 and explored associations between eating disorders and gender dysphoria. At the time of assessment, 33 participants had not begun a social gender transition, whereas 46 had completed and 18 were in the process of transitioning socially.

#### Qualitative and case report studies

Within the articles retained for the literature review, 22 utilized a qualitative or case study approach. Most were case studies (*n* = 18) detailing one to five individual cases of gender dysphoria, eating disorders, body image and dissatisfaction, and body size using a mix of qualitative and quantitative data. The findings from these case reports focused on transgender patients and their outcomes of gender affirming medical treatment [30, 48, 49], psychotherapy [50–52], psychiatric care [53, 54], eating disorder treatment [55–59], nutrition assessments [33], some combination of treatments (e.g., gender affirming medical intervention and eating disorder treatment) [60], or diagnoses (e.g., autism spectrum disorder, gender dysphoria, and eating disorder) [61]. Three case reports included detailed patient medical records [61, 62].

The four remaining articles utilized interviews with TGNB youth [7, 29, 36, 43]. These articles included adolescent and young adults with sample sizes ranging from 9 to 90. Race and ethnicity were not always reported, though when included were made up of predominately racially White participants from the United States. The interview articles with larger samples showed greater age and racial/ethnic diversity.

The description of participants’ TGNB identity, expression, or dysphoria varied across the 22 qualitative and case report studies. In some studies, participants had transitioned socially (e.g., name and sex marker changes, dress and physical presentation, etc.) or medically (e.g. gender affirming hormone therapy, gender affirming surgery, etc.) [7, 33, 48, 53, 55, 61–63], were diagnosed with gender dysphoria [7, 49, 50, 53, 54, 56–58, 60, 61], had self-identified as transgender or gender non-binary/fluid [30, 43, 48, 59, 64], or some combination of these (e.g., self-identification and gender transition). One study used “biological” male or female to describe transgender participants and did not include the self-identification of the participants [62].

### What are the risks and protective factors for eating and body image related problems?

#### Eating disorders and pattern

Prevalence of eating disorders in studies utilizing medical records identified comorbidities of eating and mental health disorders (e.g., anxiety in school age children, depression in adolescence ages) [47] and gender dysphoria [5] in transgender minor youth. Seven case studies representing 15 participants focused on eating disorders and found similar associations between gender dysphoria and mental health disorders [49, 56–58, 60, 61]. Couturier et al. [58] noted in their adolescent cases (*n* = 5), the severity of symptoms of depression and suicidality were exacerbated by delay in seeking treatment for gender dysphoria. Ristori et al. [49] utilized gender affirming hormone therapy in their case studies and found reductions in disordered eating with two transgender adolescent patients. In survey studies on eating patterns and habits comparing TGNB youth to cisgender youth, TGNB participants reported the highest levels of use of diet pills and laxatives as compared to cisgender peers [65], utilized both healthy and unhealthy eating patterns [42], and increased dieting and restrictive eating patterns were associated with weight-based victimization from peers and family [41]. Finally, autism spectrum disorder may introduce unique patterns in eating that may or may not be associated with gender dysphoria [61].

Studies analyzing only young adults largely sampled college students in the United States. These studies found that TGNB young adults were more likely to engage in disordered eating, purging, and use of diet or laxative pills than their cisgender peers [45]; and, much like with minor youth, comorbidities with mental health were more prevalent among TGNB young adults with eating disorders than their cisgender peers [46, 66]. Prevalence varied for non-binary and genderqueer college students. In 2020, Simone et al. [28] found genderqueer or gender non-conforming college students were similar to transgender and cisgender women in likelihood of disordered eating and impaired academic performance. Then in 2022, with a larger college sample of TGNB students, Simone et al. [34] found genderqueer or gender non-conforming young adults experienced the highest prevalence rates of clinically relevant eating disorder symptoms (38.8%) as compared to transgender women (37.1%), transgender men (34.0%), and gender expansive (34.1%) peers. In both studies, gender identity and eating disorder symptoms were self-reported.

In one qualitative study, a large sample of TGNB young adults (*n* = 84) differed in the degree to which they saw their disordered eating as connected to body image. Some saw clear connections where eating patterns used to change their body’s shape or size to fit a gendered ideal, while others did not see a connection [32]. The most common disordered eating pattern identified in interviews by Gordon et al. with TGNB young adults was binge eating [29]. Finally, only two studies addressed general eating patterns [42, 67]. Pistella et al. [42] explored relationships between gender identity, school safety, and weight-related behaviors among a sample of middle and high school students; they found that TGNB students reported healthier eating behaviors related to vegetable, fruit, dairy, and juice intake when their school environment was perceived as safe. Finally, Linsenmeyer et al. [67] screened adolescent and young adults visiting a gender clinic identifying 28% with possible disordered eating and 21% with possible food insecurity, which is twice the national average for the United States.

#### Body image, satisfaction, and checking

Body image was generally defined in studies (*n* = 15) as perception and feelings about one’s own physical body (e.g., appearance, maturity, and features like height, weight, and body size). In qualitative interviews, body dissatisfaction was shaped by gender dissociation, dissatisfaction with body size, and their intersections [43]. Peterson et al. [68] postulated body dissatisfaction in TGNB youth may represent a proxy for gender dysphoria. Two quantitative studies considered transgender congruence (e.g., “the degree to which transgender individuals feel genuine, authentic, and comfortable with their gender identity and external appearance,” p. 179) [69] alongside body image scales [35, 44]. In a large U.S. survey of TGNB adolescents, higher transgender congruence was negatively associated with binge eating, cognitive restraint, purging, caloric restriction, and muscle building [35].

Samples focusing exclusively on young adults included one survey with nonbinary-identified participants [27], one case study with a transgender woman exploring body image [55], one case study examining identity congruence from a psychoanalytic perspective [51], and two studies reviewing fMRI scans finding differences in the brain’s body image network for transgender individuals diagnosed with gender dysphoria [70, 71]. For nonbinary young adults, body checking and body appreciation were predictors for disordered eating patterns [27].

### What are the empirically supported treatments for eating and body image problems for TGNB patients?

No single modality for psychotherapy treatment was empirically supported in the literature, though cognitive behavioral therapy was common in case studies [56]. Gender affirming medical interventions (e.g., hormone therapy) was identified across studies as efficacious for reducing disordered eating and poor body image. Studies with minor adolescent youth and body image variables found those receiving gender affirming hormone therapy saw an improvement in their body satisfaction [72], body dissatisfaction significantly influenced quality of life [73], and suicide attempts were significantly associated with a desire for weight change [68]. Qualitatively, one study interviewing nine TGNB youth (ages 16 to 20) described the experience of gender-body *in*congruence being exacerbated when parents accessing delayed gender affirming treatment that would aid in body changes to match gender identity [7]. Delayed treatment seeking by parents was due to several reasons including lack of initial acceptance and financial constraints.

Young adult samples found higher transgender congruence and body satisfaction was associated with fewer negative mental health symptoms among those who received more gender affirming medical treatments (e.g., hormone therapy, surgery) compared to those who received less treatment or no treatment at all [44]. Five case studies including five young adult transgender women and two transgender men described the diagnoses, comorbidities, and treatments for eating disorders. All of the transgender women and one transgender man in the case studies described were using eating behaviors, such as restriction and diet pills, to change their size and body shape to meet gender ideals or delay development [48, 52, 53, 59]. In one case study, Donaldson et al. [30] reported on five TGNB patients who were receiving both gender affirming hormone therapy and in multidisciplinary eating disorder treatment, though the modality of psychotherapy was not provided. The patients varied on family support, which impacted treatment trajectory. Donaldson et al. noted the significance of family support and acceptance for retention in treatment and recovery from eating disorders.

## Discussion

The aims of the scoping review were to critically analyze all known published literature on disordered eating and body image with TGNB youth (including minor children and young adults). The review covered areas of risk and protective factors for eating disorders and body image, who is represented in the study samples, methodologies employed in the literature, and treatment modalities and associated factors. In addition, we noted bias and limitations across studies inclusive of language and its limits. The increased prevalence of eating disorders and body image related problems among TGNB youth, especially young adults, is well established through large, representative surveys with insights about causes, risks, and protective factors in case reports and qualitative interviews [5, 45, 47]. This review identified the significant overlap of mental health, eating disorders, body image, and gender dysphoria, as MST would predict, and are outlined in Table 2 of the primary research questions and associated findings.

**Table 2:** Synthesis of results based on review questions

Review question	Summary of results
Who is being included and excluded in the TGNB samples of studies on eating and body image related problems?	Predominately racially white samples though some larger U.S. studies were racially/ethnically diverse Nonbinary and gender queer youth were often not analyzed separately from transgender youth in samples Language variation about transgender identities and diagnoses
What methodologies are being used to study eating and body image related problems with TGNB people?	28 quantitative studies, all cross-sectional 4 qualitative studies using interviews 18 case studies Comorbid mental health issues measured and emphasized across study types
What are the risks and protective factors for eating and body image related problems?	Risks include delay in gender affirming medical treatment for gender dysphoria, dissatisfaction with body size related to cultural gender-body ideals, body checking, transgender congruence, family rejection Protective factors include receiving gender-affirming medical intervention, safe school environment, and family and social support
What are the empirically supported treatments for eating and body image problems for TGNB patients?	No single psychotherapy treatment modality has been tested in a clinical trial. Most treatment studies were case studies Family support and acceptance may impact treatment access, timing, trajectory, and outcome Gender affirming medical intervention was used alongside other treatment modalities for alleviating eating disorder symptomology, gender dysphoria, and body image problems

Common mental health comorbidities for minor TGNB children included anxiety [47], whereas adolescent and young adult samples reported depression, suicidal ideation and attempt, and self-injury [46, 66] with eating disorder and body image related problems. The methods used could not provide causal conclusions or offer insights into the developmental trajectory of mental health for TGNB youth from childhood to early adulthood. This is an important area for future research on etiology of mental health overall inclusive of eating and body image as it relates to other important distal factors of MST. Significant distal factors, based on this review, include family and social acceptance, timing of coming out, ability to access medical intervention (if needed) that are timely to the needs of the youth. Eleven of the studies explicitly named families and social aspects of the youth’s life [6, 7, 29, 30, 41, 43, 57, 58, 60, 62, 74] and six of those were case reports with one to five participants. Watson et al. [6] identified supportive family and friends are significant protective factors against eating disorders for transgender youth. Only two studies used theories that were inclusive of the social lives of youth [7, 29]. New applications of MST for TGNB youth describe how family acceptance of gender identity, expression, and support to seek gender affirming medical interventions are a unique feature of TGNB youth development that significantly influences mental health [9].

Thus, understanding the etiology, prevention, and treatment of eating disorders and body image problems of TGNB youth requires inclusion of family as key factors and points of interventions [75]. Future research and associated theories should be inclusive of social and family factors [9] and issues of embodiment (like objectification theory) [76] for a better understanding the interplay of eating patterns and body image. Minority stress, family, developmental, and social-ecological theories may aid in understanding the impact of external stressors, including family dependency [9], housing stability, and food insecurity [77], on eating patterns, body image, academic performance, and mental health. For example, eating disorders for some TGNB youth may be prevented through early use of puberty blockers that pause the development of secondary sex characteristics. TGNB youth noted the desire to preventing puberty through restrictive eating and the use of diet pills and laxatives [45, 65].

Many of the studies lacked diversity in other dimensions of identity or context, especially in the minor children and adolescent studies. In particular, this review noted a lack of racial and ethnic diversity in some samples, only one study exclusively focused on non-binary or gender queer individuals, and one article describing the treatment of a TGNB youth with autism spectrum disorder [60]. This may reflect high concealment given the associated risk of violence and loss of housing experienced by TGNB youth from minoritized racial/ethnic groups [78] and a lack of research focus to date on intersectionality in TGNB youth studies. Future studies taking an intersectional lens should consider implicit and explicit biases for youth from multiple marginalized groups (e.g., Black trans youth) and the significance familial and cultural contexts for shaping health [79]. For example, in some of the case reports of treatment trajectories, it seemed as if researchers and clinicians saw the TGNB identities as the core problem driving disordered eating and body image, not gender incongruence or dysphoria. This is counter to the conclusions drawn in this review. Overall, the analysis demonstrated the use of gender affirming medical interventions for creating body-gender congruence [43, 44] when in the context of family and social affirmation and support [6], allowed for treatment of eating disorder and body image problems to be addressed with associated reductions in other comorbid mental health conditions [35, 44].

From 2018–2022, 57% of the studies in this literature review were published (n = 28 studies) suggesting empirical research continues to increase for TGNB youth. Recent studies are exploring differences between transgender (transgender men/women, girls/boys) and non-binary or gender queer youth where non-binary/gender queer youth may not have the same heightened risk of poor body image when they see themselves outside of stereotypical social expectations and gender norms [61]. Though other studies find similar or higher risks for disordered eating for non-binary or gender queer youth as compared to transgender youth [34]. MST would suggest there are likely significant factors, either distal or proximal, driving within group differences that is not yet measured and considered in analysis. For example, individuals who occupy multiple marginalization groups (i.e. intersections of race/ethnicity, socioeconomic level, education) and have low social and family acceptance [80] likely experience differential risks for disordered eating regardless of gender identity. Within group differences will be useful to substantiate for informing improved treatment modalities and approaches.

There is an inherent limitation to estimating eating disorder and disordered eating prevalence in that only one instrument has been tested for use with TGNB youth at this time [68]. TGNB youth may utilize eating or exercise behaviors for purposes distinct from their cisgender peers such as: weight manipulation for a body size or shape that better aligns with one’s gender identity [26]; suppression of pubertal development and secondary sex characteristics (e.g., voice changes, development of chest tissue, etc.); menstrual suppression [26]; masking of body features that do not align with gender identity; and as a coping mechanism for minority stressors. Validation of existing measures commonly used to screen for eating disorders and body image with TGNB youth should be inclusive of differences for nonbinary and gender queer youth and based on development age groups—minor children, adolescent, and young adults—and their goals for creating body-gender congruence.

## Limitations

This review has several limitations. The team carefully planned and utilized software to accurately answer the study questions and conduct the review. However, research studies may have been missed. The current studies still lack nuance by variations in gender identity, developmental age, expression, race, neurodiversity, and social factors. Limiting the search to English means other international studies were missed. Some of the studies included transgender and non-transgender samples, requiring reliance on portions of the data or only descriptive analysis. Many of the studies were cross-sectional in nature, limiting causal associations between risk factors, treatments, and outcomes documented in the studies. Finally, the rigor could have been enhanced by pre-registering our search protocol with the International Prospective Register of Systemic Reviews.

## Conclusion

The scoping review offers an overview and critical examination of research with TGNB youth who experience eating and body image related problems as well as clinical studies on treatment approaches and effectiveness. The 49 studies identified demonstrated the prevalence of eating disorder and body image related problems for TGNB youth as compared to their peers. Future research should intersectional approaches to treatment that allow for increased racial/ethnic diversity, the co-occurrence of neurodiversity (e.g., autism), and family and social factors influencing eating patterns, body image, mental health, and treatment outcomes.

## Data Availability

Search terms and data retrieved through library searches are available upon request to the corresponding author.
